# Encapsulation and Ultrasound-Triggered Release of G-Quadruplex DNA in Multilayer Hydrogel Microcapsules

**DOI:** 10.3390/polym10121342

**Published:** 2018-12-05

**Authors:** Aaron Alford, Brenna Tucker, Veronika Kozlovskaya, Jun Chen, Nirzari Gupta, Racquel Caviedes, Jenna Gearhart, David Graves, Eugenia Kharlampieva

**Affiliations:** 1 Department of Chemistry, University of Alabama at Birmingham, Birmingham, AL 35294, USA; aaaron@uab.edu (A.A.); batucker@uab.edu (B.T.); vkozlovs@uab.edu (V.K.); cj1016@uab.edu (J.C.); nirzari@uab.edu (N.G.); racquelc@uab.edu (R.C.); gea7026@student.waynesburg.edu (J.G.); dgraves@uab.edu (D.G.); 2 Center of Nanoscale Materials and Biointegration, Birmingham, AL 35294, USA

**Keywords:** multilayer capsules, layer-by-layer, DNA, ultrasound, microcapsules, hydrogen-bonded, hydrogel

## Abstract

Nucleic acid therapeutics have the potential to be the most effective disease treatment strategy due to their intrinsic precision and selectivity for coding highly specific biological processes. However, freely administered nucleic acids of any type are quickly destroyed or rendered inert by a host of defense mechanisms in the body. In this work, we address the challenge of using nucleic acids as drugs by preparing stimuli responsive poly(methacrylic acid)/poly(*N*-vinylpyrrolidone) (PMAA/PVPON)_n_ multilayer hydrogel capsules loaded with ~7 kDa G-quadruplex DNA. The capsules are shown to release their DNA cargo on demand in response to both enzymatic and ultrasound (US)-triggered degradation. The unique structure adopted by the G-quadruplex is essential to its biological function and we show that the controlled release from the microcapsules preserves the basket conformation of the oligonucleotide used in our studies. We also show that the (PMAA/PVPON) multilayer hydrogel capsules can encapsulate and release ~450 kDa double stranded DNA. The encapsulation and release approaches for both oligonucleotides in multilayer hydrogel microcapsules developed here can be applied to create methodologies for new therapeutic strategies involving the controlled delivery of sensitive biomolecules. Our study provides a promising methodology for the design of effective carriers for DNA vaccines and medicines for a wide range of immunotherapies, cancer therapy and/or tissue regeneration therapies in the future.

## 1. Introduction

Nucleic acids are the universal biological code and dictate the essential processes in all organisms. Owing to their biological relevance, thorough characterization including everything from fundamental structural studies [1,2] to their potential use in a variety of applications has been reported [3,4,5]. In recent years, the delivery of therapeutic nucleic acids to modify gene expression has emerged as a promising therapeutic method for a range of inherited or acquired diseases [6,7,8]. Among the multitude of repeating nucleic acid motifs, the human G-quadruplex sequence has emerged as a potentially relevant key to new therapeutic possibilities [9]. The G-quadruplex offers unique DNA structures formed from guanine-rich sequences of DNA that can be found within promotor regions of oncogenes as well as in the telomeric regions of the chromosome [10,11]. G-quadruplex structures have particular conformational stabilities that can be observed due to their tendency for the unique secondary structure to assemble from multiple or single-strands of DNA if the repeating GGG motif spans four closely interspersed sequences [12,13]. The G-quadruplex structural motif is nuclease resistant, plays a role in transcriptional regulation, and is structurally similar to the thrombin binding aptamer (TBA) [14,15], which is itself a G-quadruplex forming a sequence of DNA that is commonly used as an anti-coagulant during surgeries [16,17]. As G-quadruplex formations can be found in the telomeric chromosomal region, and chromosomes with damaged or shortened telomeres can cause miscoding that may result in cancerous cells [9], delivery of G-quadruplex DNA is being investigated as a component of gene-mediated anticancer treatment [18,19]. Despite their potential, nucleic acid therapeutics as disease treatment strategies have not been widely adopted due to the series of extracellular and intracellular barriers preventing effective delivery of nucleic acids to the desired sites [7]. When free DNA is administered as a therapeutic agent, it can be rapidly degraded by nucleases in the plasma or the extracellular matrix [20]. In addition, non-degraded DNA is rapidly cleared from circulation with high likelihood [21], while the lack of endosomal escape, degradation by lysosomes [22], and insufficient cytosolic transport represent more specific obstructions to therapeutic efficacy if the DNA can be internalized by cells [23,24]. In response to these challenges, both viral- and non-viral vectors have been suggested as a way to increase therapeutic potential by providing assistance for intracellular transport and release of DNA at the target site [25,26]. The promising natural efficiency of viral vectors has encouraged recent studies on their development. However, several limitations have been associated with the use of viral vectors including low capacity, challenges in scale-up, high cost, and risk of mutagenesis [27,28]. Furthermore, vector-inactivating immune responses are frequently observed with repeated administrations or prior exposures, which limits the efficacy of these drugs [7]. Non-viral vectors synthesized from a variety of different cationic lipids or polymers can condense the DNA into micro- or nanoparticles and offer a reprieve from the limitations of viral vectors [24]. While synthetically simple and commercially available, cationic non-viral vectors such as Lipofectamine 2000 and polyethyleneimine (PEI) have been shown to cause cytotoxicity [24,29], and it has also been discussed that the direct conjugation of non-viral vectors to DNA may change the native properties and functions of the nucleic acid sequence [30]. Furthermore, while liposomal and microbubble-based delivery systems have been shown to utilize ultrasound (US) to release encapsulated gene therapeutics [31,32], these strategies still suffer from low release efficiency and inconsistent results [33]. Consequently, it is of significant importance to develop a safe and biocompatible non-viral vector that can encapsulate DNA inside an inert carrier without changing the structural integrity of the DNA while still offering an effective, controllable release mechanism.

The layer-by-layer (LbL) assembly of polymers at surfaces has widely been demonstrated as a useful method to engineer thin coatings, hollow capsules, and network hydrogels for various therapeutic and preventative strategies owing to the nanoscale control over thickness, architecture, and chemistry of the materials [34,35]. The LbL assembly allows for the design of hybrid materials that incorporate both synthetic and biomolecules in their architecture. For example, Lvov et al. demonstrated that negatively charged DNA could be incorporated into LbL multilayers by electrostatic interactions with positively charged cationic polymers as building blocks [36,37,38]. The ability of LbL multilayers to not only incorporate but also release potentially therapeutic DNA has been the focus of recent studies [39]. Lynn et al. has recently shown the release of plasmid DNA from LbL multilayers via film erosion or contact transfer from mixed multilayers of ester and charge transfer polymers, or electrostatically-assembled systems of poly(acrylic acid)/polyethyleneimie (PAA/PEI), respectively [40,41]. The Hammond group has also shown multilayer coatings for the delivery of CTGF (connective tissue growth factor)-silencing siRNA with the aim of reducing scar formation in burn treatments [42]. While the studies mentioned above showcase the remarkable ability of LbL systems to incorporate and release DNA through a variety of novel pathways, they are focused on flat 2D films and surface coatings, which may not translate to therapeutic strategies involving injected therapeutic agents that reach targets in the body through circulation.

In response to this limitation, 3D micro- and nanoparticles that incorporate therapeutic DNA have been the subject of recent development in LbL systems [43,44]. These LbL-assembled polymer microcapsules are promising non-viral vectors for drug delivery due to their biocompatibility, micron-sized interior cavity which enables high loading capacity, and potential for targeted delivery of their cargo [30,45,46,47], having been used as model vehicles for encapsulation of DNA within the hollow cavity or the shell of the capsule [48,49]. Through selection of polymer type, molecular weight, and crosslink chemistry, the nanothin shells of LbL hydrogel microcapsules can be tailored for controlled cargo release via stimuli including pH, temperature, and US irradiation [50,51,52]. The LbL assembly process also enables association between the capsules and their cargo as well as the type and degree of response to stimuli [53]. Well-documented strategies by Caruso et al. and Sukorukhov et al. approach incorporating DNA within the interior of degradable microcapsules by loading DNA into calcium carbonate microparticles followed by coating of LbL multilayers with enzyme degradable crosslinks and dissolution of the inorganic templates [54,55,56,57]. A recent report has also described delivery of plasmid DNA via passive transfection of DNA-loaded LbL silk microparticles into NIH/3T3 fibroblasts [58].

Though the feasibility of DNA encapsulation is evident from the above studies, they have mostly been focused on enzymatic degradation, which may be limited by the conditions of the target environment. Therefore, the encapsulation and efficient multiresponsive release of a promising DNA target such as G-quadruplex-forming sequences via enzyme and US-mediated capsule destruction represents a significant advancement in the field of controlled delivery of gene therapeutics but has yet to be reported in the field. In addition, conservation of the critically-important secondary structure of DNA after undergoing the conditions of the release mechanism has yet to be demonstrated. Finally, while enzyme-triggered release has been demonstrated to be efficient, gene vectors that are responsive to US in addition to intracellular redox environments offer the best approach to improving overall efficacy, as it has been widely demonstrated that unfavorable cell membrane crossing of microparticles and small drugs can be greatly assisted by the US-exclusive sonoporation effect [59,60,61,62].

We have recently reported that PMAA hydrogel particles crosslinked with the enzyme-degradable crosslinker cystamine can release the anticancer drug doxorubicin (DOX) upon introduction of intracellular concentrations of glutathione (GSH) [63,64]. We have also shown with hydrogen-bonded tannic acid/poly(*N*-vinylpyrrolidone) (TA/PVPON) multilayer microcapsules that the assembly conditions and US dose can be customized to tailor their susceptibility to US irradiation, releasing the same drug in sustained or burst-type dosing in vitro and in vivo [65,66]. US has been widely used in gene delivery because the stimulation offers a traceable, localized and non-invasive way to release therapeutic cargos. More importantly, US has been shown to assist in brain uptake of DNA [67], enhance microbubble mediated gene delivery [68], and increase the permeation of therapeutics into target cells [69,70]. In addition to the type of stimulus applied to microcapsule drug carriers, an important parameter controlling the encapsulation and release of therapeutic cargo is the pore size of the shell network. Regarding this, we have shown that hollow microcapsules with shells made of interpenetrated networks of PMAA/PVPON are capable of efficiently loading and releasing hydrophilic model therapeutics from among a wide range of molecular weights [71]. While we demonstrated the use of this new system for low molecular weight (*M*_w_ < 1000 Da) small molecules, we now show that the microcapsules with interpenetrated PMAA/PVPON shell networks allow efficient loading and precisely controlled release of nucleic acid oligomers and longer double stranded sequences.

In the current work, we study the conditions for fabrication of PMAA/PVPON multilayer hydrogel microcapsules using porous CaCO_3_ sacrificial templates as well as the encapsulation and controlled release of two types of nucleic acids (G-quadruplex and double-stranded calf thymus) through US irradiation and GSH exposure. We show that the (PMAA/PVPON) multilayer hydrogel capsules can encapsulate and release both ~450 kDa double stranded DNA and shorter ~7 kDa G-quadruplex oligonucleotides. The (PMAA/PVPON) multilayer hydrogel was chosen due to the biocompatibility of the polymers used for fabrication of various drug carrier constructs [63,72,73,74]. To our knowledge, this is the first example of multi-responsive multilayer microcapsules with the capability of G-quadruplex DNA delivery and preservation of the critical secondary structure after release. Our approach may provide a promising methodology for the design of effective carriers for DNA vaccines and medicines for a wide range of immunotherapies, cancer therapy and/or tissue regeneration therapies in the future.

## 2. Materials and Methods

### 2.1. Materials

Poly(methacrylic acid) (PMAA, average *M*_w_ 100,000 Da), poly(*N*-vinylpyrrolidone) (PVPON, average *M*_w_ 1,300,000 Da), poly(ethyleneimine) (PEI; average *M*_w_ 70,000 Da), l-glutathione reduced (GSH), and cystamine dihydrochloride (CS) were purchased from Sigma-Aldrich (St. Louis, MO, USA). 1-ethyl-3-(3-(dimethylamino)propyl)-carbodiimide hydrochloride (EDC) was purchased from Chem-Impex International (Wood Dale, IL, USA). Monobasic sodium phosphate (NaH_2_PO_4_), dibasic sodium phosphate (Na_2_HPO_4_), ethylenediaminetetraacetic acid sodium salt (EDTA), sodium chloride (NaCl), calcium chloride (CaCl_2_), sodium carbonate (Na_2_CO_3_), ethidium bromide, and calf thymus DNA (ctDNA) were purchased from Fisher Scientific (Hampton, NH, USA). DNA oligonucleotides (5’-AGGGTTAGGGTTAGGGTTAGGG-3’) were purchased from Midland Certified Reagents (Midland, TX, USA).

### 2.2. Preparation of Double-stranded Calf Thymus DNA

Calf thymus DNA was dissolved in 0.01 M NaH_2_PO_4_, 0.01 M Na_2_HPO_4_, 0.001 M EDTA (BPE) buffer at 4 °C overnight before sonication using a Branson sonifier (Danbury, CT, USA) for 5 min on/off intervals for a total of 30 min. The DNA was kept over an ice bath and nitrogen gas was bubbled through the sample throughout the sonication process. A syringe filter (0.45 μm) was used to filter the sonicated solution and the salt concentration was increased to 0.2 M by the addition of NaCl. The DNA sample was treated with RNase at 37 °C for 30 min followed by treatment with proteinase K at 37 °C for 2 h. The DNA sample was extracted with an equal volume of chloroform in a separatory funnel before being split into two 50-mL Falcon tubes. The tubes were centrifuged at 4 °C for 5 min and the top layers from each tube were added back to the separatory funnel. This process was repeated until the interface between the DNA solution and the chloroform was clear. The DNA layers were then pooled together and precipitated by adding salt-saturated ethanol to the solution. The precipitated DNA was separated by centrifugation and dried in vacuo. The resulting DNA pellet was dissolved in BPES buffer (0.01 M NaH_2_PO_4_, 0.01 M Na_2_HPO_4_, 0.001 M EDTA, 0.150 M NaCl) and dialyzed against BPES buffer. After a minimum of 4 buffer changes, the DNA was collected from dialysis, filtered again using a 0.45 μm syringe filter, and stored at 4 °C for future use. The average length of the calf thymus DNA was determined by an agarose gel to be approximately 700 base pairs, or ~450 kDa. The concentration of the DNA sample per duplex was determined by monitoring the UV–Vis absorbance at 260 nm (ε = 13,200 M^−1^·cm^−1^ (base pairs)).

### 2.3. Preparation of DNA Oligonucleotides

G-quadruplex forming oligonucleotides were resuspended in sodium phosphate buffer (50 mM NaH_2_PO_4_, 50 mM Na_2_HPO_4_, 150 mM NaCl, and 0.01 mM EDTA) and stored at 4 °C overnight. The G-quadruplex was allowed to form by heating the dissolved DNA to 90 °C and allowing it to cool at a rate of 0.1 °C per minute to ambient temperature before refrigeration. The concentration of DNA per strand of G-quadruplex was assessed by measuring the absorbance at 260 nm at room temperature (ε = 228,500 M^−1^·cm^−1^ (strand)). When not in use, oligonucleotide solutions were stored at 4 °C.

### 2.4. Encapsulation of DNA into Calcium Carbonate Microparticles

G-quadruplex oligonucleotides were encapsulated into porous calcium carbonate microparticles by combining 150 µL CaCl_2_ solution (1 M), 1190 µL ultrapure water, and 10 µL of 1 mM DNA solution with 150 µL Na_2_CO_3_ (1 M) under rapid stirring for 40 s. G-quadruplex-loaded microparticles were removed from the stir plate, centrifuged to remove the supernatant, and washed by adding ultrapure water and vortexing the sample. The G-quadruplex-loaded cores were washed with water a second time and examined with optical microscopy to determine their size and ensure they had not fused or aggregated. The supernatants from the wash step were saved for further analysis of the capsule loading capacity of G-quadruplex. Empty cores were also prepared using this same strategy.

Double stranded calf thymus DNA was encapsulated by mixing 450 µL CaCl_2_ (1 M), 2.07 mL ultrapure water, and 450 µL ctDNA (6.1 mM). Once this solution spun uniformly, 450 µL Na_2_CO_3_ (1 M) was added and after 40 s, the cores were collected and washed as described above. To make the control (empty) calcium carbonate cores for this system, 450 µL of sodium phosphate buffer was added instead of the ctDNA.

### 2.5. Synthesis of G-Quadruplex-Loaded (PMAA/PVPON)_n_ Multilayer Hydrogel Capsules

The spherical DNA-loaded calcium carbonate templates were shaken with an aqueous solution of 1 mg/mL PEI to assist entrapment of DNA and to enhance binding of the polymers to the surface. Alternating adsorption of PMAA or PVPON from polymer solutions (0.25 mg/mL in 0.01 M PO_4_ at pH 4) was performed starting from PMAA with 10 min-deposition time followed by centrifugation to exchange the supernatant for buffer and rinse nonadsorbed polymer. After triplicate rinsing, PVPON solution was added and the deposition process was repeated until the desired number of (PMAA/PVPON) polymer bilayers had been deposited. After complete polymer deposition, (PMAA/PVPON) core-shells were allowed to shake with 1-ethyl-3-(3-(dimethylamino)propyl)-carbodiimide hydrochloride (EDC) (5 mg/mL, pH 5.5) for 30 min before being crosslinked with cystamine dihydrochloride (5 mg/mL, pH 5.5) for 48 h. The cross-linked core-shells were washed twice with buffer (NaH_2_PO_4_, pH 5.5) before EDTA (1 mM, pH 5.5) was added to dissolve the cores. The dissolution of the CaCO_3_ templates was followed via optical microscopy and scanning electron microscopy (SEM). Once the spherical templates had dissolved, the (PMAA/PVPON)_n_ capsules were washed and dialyzed against 0.01 M phosphate buffer (pH 7.4). After 72 h, capsules were removed from dialysis, washed with phosphate buffer (pH 7.4), and stored for study.

### 2.6. Characterization of (PMAA/PVPON)_13_ Capsules Using Fourier Transform Infrared Spectroscopy (FTIR)

FTIR was used to analyze the chemical composition of PMAA/PVPON capsules. Empty and G-quadruplex-loaded (PMAA/PVPON)_13_ hydrogel capsules at pH 7.5 as well as G-quadruplex forming oligonucleotides at pH 7.5 were used to assess the location of nucleotides in the capsule shell. To prepare the samples for FTIR, 0.5 mL of each at pH 7.5 were freeze-dried overnight. Spectra were collected in absorbance mode using a Bruker Alpha-FTIR (ATR-FTIR) (Billerica, MA, USA). The number of scans collected for each of the background and sample measurements equaled 128. The resulting spectra were normalized and baseline corrected.

### 2.7. Characterization of (PMAA/PVPON)_n_ Capsules by SEM and Confocal Laser Scanning Microscopy (CLSM)

A FEI Quanta FEG SEM microscope (Hillsboro, OR, USA) at 10 kV was used to image dried suspensions of G-quadruplex-loaded (PMAA/PVPON) capsules. Capsules were dialyzed against DI water to prepare them for SEM, and a drop of the diluted capsule solution was placed on a silicon wafer and allowed to dry overnight. A Denton sputter-coater was used to coat the dried capsule samples with a ~5 mm thick layer of silver immediately before imaging. For CLSM imaging, 30 µL of the 1.6 × 10^8^ capsules/mL stock solution was pipetted into Lab-Tek confocal microscopy (EMS, Hatfield, PA, USA) chambered coverglass containing buffer solutions. A 10 µL aliquot of Alexa Flour 568 hydrazide (0.5 mg/mL) fluorescent dye solution was added to assist in visualizing the capsules and the capsules were allowed to settle overnight before imaging with a Nikon A1R multiphoton confocal microscope (Tokyo, Japan) equipped with a 60× oil immersion objective.

### 2.8. ζ-potential Measurements of (PMAA/PVPON)_n_ Multilayer Hydrogel Capsules

A Malvern Nano Zetasizer (Spectris, Egham, United Kingdom) was used to measure the ζ-potential of the G-quadruplex-loaded (PMAA/PVPON)_n_ capsules in the presence of 0.01 M phosphate buffer solutions with pH values from pH = 3 to 7.4. The capsules were allowed to equilibrate for 10 min before being vortexed and inserted into the sample chamber. The ζ-potential was determined by taking the average of three sets of 20 individual measurements.

### 2.9. US-Controlled Release of G-Quadruplex DNA from the (PMAA/PVPON)_n_ Capsules

G-quadruplex-loaded (PMAA/PVPON)_n_ capsules with the bilayer number of *n* = 13 or 6 were burst using a Fisher 120 sonic dismembrator at 22 kHz, 14 or 55 W/cm^2^ in 20 s bursts. The sonication probe was inserted into the capsule solution and moved throughout the volume of the centrifuge tube throughout the 20-s treatment. The capsule fragments were centrifuged for 15 min at 8000 rpm, and the supernatant removed for UV spectroscopic analysis using the absorbance wavelength of 260 nm. Water and 70% ethanol were used to wash the sonication probe before and after use.

### 2.10. Turbidimetry of GSH-Treated (PMAA/PVPON)_13_ Capsules

Turbidity measurements were performed to track the GSH-induced degradation of (PMAA/PVPON)_13_ capsules at 37 °C using fluorescence spectroscopy (Varian, Palo Alto, CA, USA, Cary Eclipse). The scattering intensity of the capsules suspended in phosphate buffer (2 × 10^5^ capsules/μL) at pH 7.4 with or without 5 mM GSH was measured at λ = 700 nm [75]. The ratio of the particle scattering intensity at a certain interval to that of the initial nondegraded particles was used to calculate the relative turbidity.

### 2.11. Quantification of G-Quadruplex DNA Release

Prior to the US/enzyme treatment, the capsules were centrifuged (8000 rpm for 30 min) and the supernatant was collected, and monitored for absorbance at 260 nm with a Varian Cary 50 UV–Vis spectrometer (Palo Alto, CA, USA) in a quartz cuvette to determine any initial amounts of G-quadruplex present, and then returned to the capsules. After US/enzyme treatment, the release of G-quadruplex was determined using the absorbance at 260 nm and the molar absorptivity coefficient (228,500 M^−1^·cm^−1^ (strand)) of the G-quadruplex oligomers.

### 2.12. Quantification of dsDNA Release

The amount of dsDNA released from the capsules was determined through fluorescence measurements of DNA-loaded capsules after US treatment. Samples were excited at 520 nm and the emission spectrum was recorded from 530 to 750 nm. A 1 mm × 1 mm square cuvette was used for all measurements and a 1% *v*/*v* amount of ethidium bromide (EtBr) was added to each sample before collecting the emission data. Samples equilibrated for 10 min before data collection. A calibration curve was collected for systematically increasing amounts of dsDNA and the emission at 605 nm was used to calculate the dsDNA concentration in each sample.

### 2.13. Probing Structural Changes of G-Quadruplex DNA after Release

DNA released from the capsules was examined for any changes to the secondary structure using circular dichroism (CD) spectroscopy. CD scans of free G-quadruplex were compared to released DNA from both the CaCO_3_ cores and the (PMAA/PVPON)_n_ capsules to determine any changes to the secondary structure. Free and released G-quadruplex scans were obtained from an average of 3 scans collected from 320–220 nm with a 1-nm step and a 1-s response time.

## 3. Results and Discussion

### 3.1. Synthesis of (PMAA/PVPON)_n_ Hydrogel Capsules Using Calcium Carbonate Sacrificial Templates

**Figure 1:**
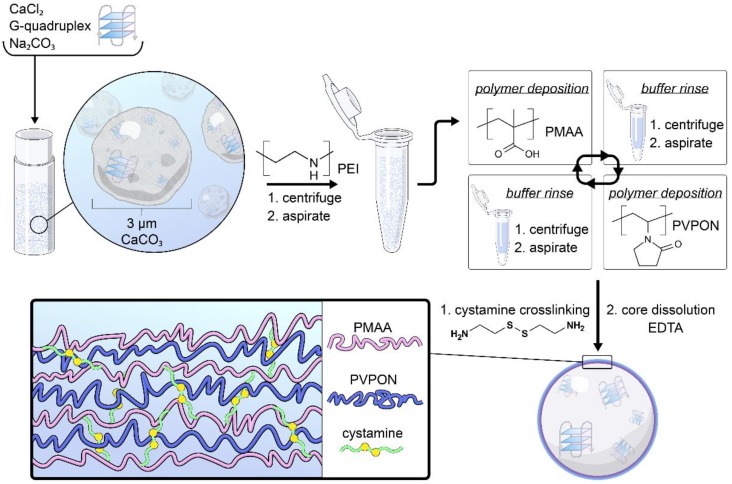
Fabrication of the (PMAA/PVPON)_n_ multilayers. The DNA-loaded (PMAA/PVPON)_n_ microcapsules were prepared by co-precipitation of Na_2_CO_3_ and CaCl_2_ salts with G-quadruplex or dsDNA in aqueous solution. PEI was adsorbed onto particle surfaces to increase interaction with the first PMAA layer. PMAA and PVPON were alternatingly adsorbed onto particles surfaces until the desired number of bilayers (n) was formed. The core-shells were crosslinked with cystamine before dissolving the sacrificial template in EDTA, resulting in hollow DNA-loaded multilayer hydrogel microcapsules.

One of the characteristic properties of PMAA multilayer hydrogels is their ability to undergo dramatic volume transitions upon ionization of the PMAA carboxylates and subsequent influx of counterions. We have previously shown that both (PMAA)_n_ multilayer hydrogel particles and hollow multilayer hydrogel capsules can swell up to two times their size [51,63,74] in response to pH change from the isoelectric point of the crosslinked network (~pH = 5) to pH = 7.4, in addition to mild swelling at lower pH due to single-side reacted free amine groups from the diamine crosslinker. In these previous studies, complete removal of PVPON was ensured to achieve a single component PMAA multilayer hydrogel. However, the presence of PVPON in the (PMAA/PVPON) crosslinked multilayer and the additional chain entanglement between PMAA and PVPON components can suppress the swelling of the PMAA network. We demonstrated in a recent study that increasing the molecular weight of PVPON within cross-linked PMAA multilayer hydrogel films obtained by spin-assisted multilayer assembly decreases the ability of the PVPON to be released from the network after the pH is increased above the p*K*_a_ of PMAA [76]. Specifically, when the molecular weight of PVPON in PMAA/PVPON hydrogen-bonded multilayer template films was increased to 1,300,000 Da (1300 kDa), only 18% of the PVPON content was released due to the increased chain entanglement in comparison to shorter (58 kDa) PVPON polymer chains [76]. In another recent study, we have demonstrated that hydrogel capsules made of interpenetrated (PMAA/PVPON) multilayers can be obtained by dipped multilayer assembly of PMAA (*M*_w_ = 150,000 Da) and PVPON (*M*_w_ = 1,300,000 Da) on porous and solid silica microparticles followed by chemical crosslinking of PMAA [71]. Because of its high molecular weight, PVPON was physically entrapped within the PMAA network and did not diffuse out from the network at pH = 8 [71]. In our current work, we utilized high molecular weight PMAA (100 kDa) and PVPON (1300 kDa) to create capsules of (PMAA/PVPON)_n_ interpenetrated multilayer hydrogel using sacrificial porous calcium carbonate particles with the aim of increasing the retention of loaded DNA molecules. The subscript *n* denotes the number of polymer bilayers. Figure 1 shows a schematic overview of the synthesis of nucleic acid-loaded capsules.

**Figure 2:**
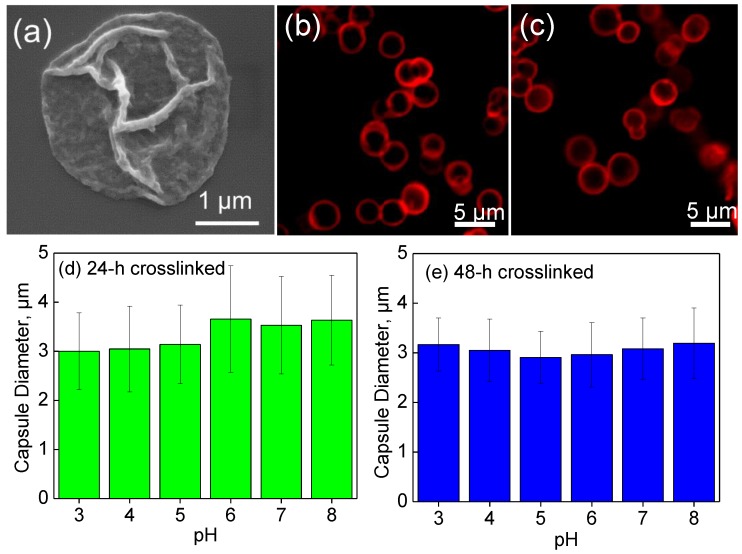
(**a**) SEM image of (PMAA/PVPON)_13_ multilayer hydrogel capsules. CLSM images of (PMAA/PVPON)_6_ hydrogel capsules crosslinked for (**b**) 24 h and (**c**) 48 h imaged in solution at pH 4. Average diameters of the capsules crosslinked for 24 (**d**) and 48 (**e**) hours as a function of pH as obtained from analysis of their CLSM images using ImageJ software (version 1.52e, NIH, Bethesda, MD, USA). The data is presented as mean ± standard deviation (SD). For each measurement series, at least 30 capsules were analyzed for each pH value.

Complete dissolution of the calcium carbonate cores and the presence of the unruptured polymer shell were confirmed by SEM. Figure 2a demonstrates the dehydrated hollow capsule shell collapsed on the surface of silicon wafer and was devoid of any fragments of the inorganic core template. Figure 2b,c show confocal microscopy images of (PMAA/PVPON)_6_ hydrogel capsules crosslinked with cystamine for 24 h (Figure 2b) and 48 h (Figure 2c) and the average diameters of the capsules at pH values increasing from pH = 3 to pH = 8 are shown in Figure 2d,e.

CLSM analysis in Figure 2d,e shows that the average size of the crosslinked (PMAA/PVPON)_13_ hydrogel capsules remained unchanged when the 24-h and 48-h crosslinked capsules were exposed to solutions with pH values increasing from pH = 3 to pH = 8. For example, the average size of the capsules crosslinked for 24 h was 3.0 ± 0.5 and 3.6 ± 0.7 µm, at pH = 3 and pH = 8, respectively. As reported previously, the cystamine-crosslinked PMAA network demonstrated pH-dependent swelling at pH > 6 and pH < 5 because of ionization of carboxylic and amine groups, respectively, similar to that observed for PMAA multilayer hydrogels cross-linked with ethylenediamine [34,63]. However, we have shown earlier that the entrapment of PVPON within the capsule wall as well as a high crosslink density can drastically change the pH-swelling profile of (PMAA/PVPON) hydrogels [71]. Thus, the presence of long PVPON chains highly entangled within the PMAA capsule hydrogel shell was shown to suppress mobility of PMAA segments between the crosslinks.

Conversely, the pH-induced swelling of (PMAA/PVPON) hydrogel capsules was shown to be almost independent of capsule crosslinking time because the swelling was mostly controlled by the presence of the PVPON [71]. Indeed, the capsule swelling suppression by entangled PVPON chains was observed in this work when more tightly crosslinked capsules (48-h crosslinked (PMAA/PVPON)_13_) were exposed to solutions of various pH values. Figure 2e shows that the average capsule size of 48-h crosslinked (PMAA/PVPON)_13_ hydrogel shells was 3.2 ± 0.8 and 3.2 ± 0.9 µm when exposed to pH = 3 and pH = 8, respectively. This data indicate that extensive crosslinking of the PMAA network resulted in entrapment of the PVPON chains already after the 24-h crosslinking time. Despite that, the 48-h crosslinked capsules were chosen for loading of nucleic acid molecules as the lowered mesh size of the network afforded by entrapped PVPON and a higher crosslinking density can also help to constrain the permeation of DNA through the shell in the absence of release stimuli.

### 3.2. Encapsulation of Nucleotides into (PMAA/PVPON)_n_ Hydrogel Capsules

**Figure 3:**
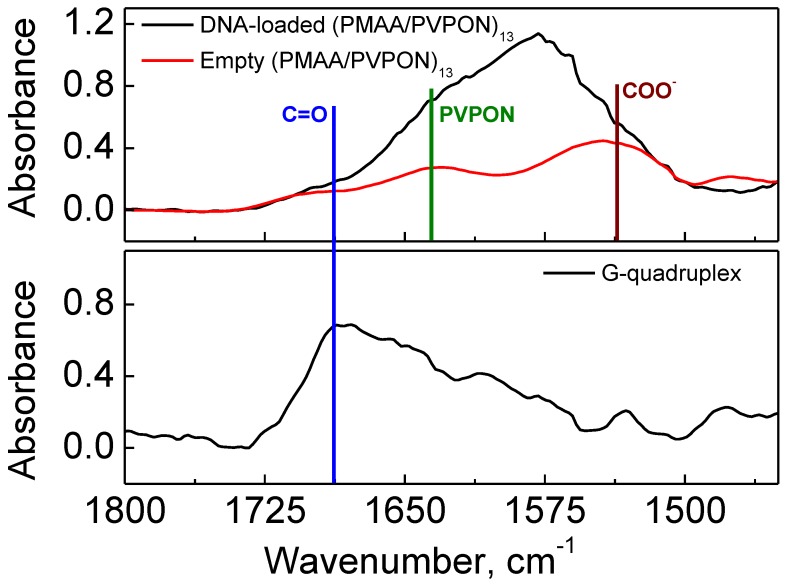
FTIR spectra of (top; red trace) empty and (top; black trace) G-quadruplex DNA-loaded (PMAA/PVPON)_13_ multilayer hydrogel capsules; and (bottom; black trace) non-encapsulated G-quadruplex oligonucleotides.

FTIR analysis was used to determine chemical composition of the hydrogel capsules after dissolution of calcium carbonate cores. For that, both empty (control) and G-quadruplex-loaded (PMAA/PVPON)_13_ hydrogel capsules were lyophilized from solution at pH = 7.4 before analysis. Figure 3 shows that the FTIR spectrum for the empty (PMAA/PVPON)_13_ hydrogel capsules (top panel; red trace) reveals major peaks centered around 1525 cm^−1^ and 1640 cm^−1^ corresponding to the presence of both the ionized carboxylate groups from PMAA (1550 cm^−1^) and the carbonyl groups of PVPON (1645 cm^−1^) [77]. The presence of the carbonyl stretch from the lactam ring in PVPON indicates that PVPON is not released from the PMAA network after crosslinking and dissolution of the cores, and remains trapped within the PMAA network. The entrapment of PVPON within the PMAA network is favored by the hydrogen bonding interactions between PMAA and PVPON layers at the crosslinking reaction condition (pH 5–5.8), which is close to that of the p*K*_a_ of PMAA (~5) [35].

Interestingly, when the capsules were loaded with G-quadruplex DNA, a broadening of the spectral peaks occurred as can be seen in the top panel in Figure 3 (black trace). The FTIR spectrum for the G-quadruplex-loaded capsules contains discernable shoulders, and the presence of PMAA and PVPON can still be detected at 1550 and 1645 cm^−1^, respectively, within the broadened peak. To discern the reason for this peak broadening, G-quadruplex-forming oligonucleotides were also freeze-dried at pH = 7.4 and analyzed with FTIR as shown in Figure 3 (bottom panel). The spectrum for the free oligonucleotide showed a broad peak centered around 1700 cm^−1^ corresponding to carbonyl groups in the guanine bases of the DNA [78]. This peak can also be seen in Figure 3 (top panel) in the black trace for the G-quadruplex-loaded capsules.

**Figure 4:**
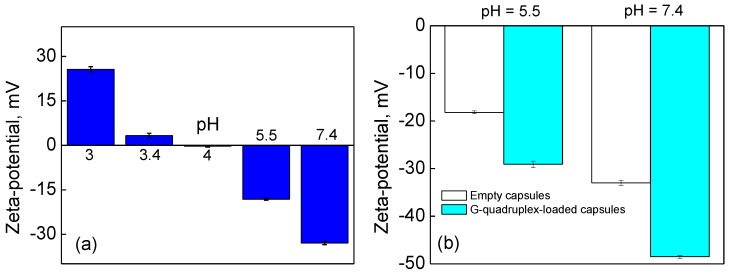
pH-Dependence of zeta-potential values of the (PMAA/PVPON)_13_ hydrogel capsules (**a**) without G-quadruplex DNA and (**b**) loaded with G-quadruplex DNA. The data is presented as mean ± SD.

We also found that some of encapsulated G-quadruplex DNA is also present within the (PMAA/PVPON) hydrogel shell. Figure 4 shows the pH-dependent changes of the capsule surface charge. At pH = 7.4, the empty (PMAA/PVPON)_13_ hydrogel capsules exhibit a negative zeta-potential of −32 ± 1 mV, while at pH = 3 the zeta-potential is completely reversed to a positive value of 25 ± 2 mV (Figure 4a). At pH = 4, the overall capsule surface charge becomes neutral, where the negatively charged ionized carboxylic groups are counteracted by protonated positively charged amine groups from single-side reacted amine crosslinks within the PMAA network as we showed earlier [63]. At higher pH values, the carboxylic acids are ionized to carboxylates consistent with the observed negative zeta-potential, which is in agreement with our previously reported data [63]. Figure 3b compares the pH-dependent capsule surface charge at the biologically relevant pH values of 5.5 and 7.5 for both empty and G-quadruplex-loaded capsules measured at the same concentration of the capsule solutions. Both types of capsules possess a negative surface charge at pH = 5.5 and pH = 7.5, with the zeta-potential values of −18.2 ± 0.3 mV and −33 ± 1 mV for the empty (PMAA/PVPON)_13_ hydrogel capsules at pH = 5.5 and pH = 7.5, respectively; and of −29.1 ± 0.7 mV and −48.5 ± 0.3 mV for the G-quadruplex-loaded (PMAA/PVPON)_13_ hydrogel capsules at pH = 5.5 and pH = 7.5, respectively (Figure 4b). However, capsules loaded with G-quadruplex oligonucleotides increase the magnitude of the negative zeta-potential (1.6-fold at pH = 5.5 and 1.5-fold at pH = 7.5) at both pH values compared to the empty capsules at the same concentration of capsules. DNA is negatively charged at neutral pH due to the phosphate ions comprising its backbone and the magnitude of this charge is influenced by salt concentration [79]. Because the solutions contain no added salt, the G-quadruplex oligonucleotides in this work are negatively charged at the pH ranges shown in Figure 4b. It is also established that the p*K*_a_ of the individual nucleotide bases are largely overwhelmed by the phosphate p*K*_a_ (~1) when they are assembled into DNA strands and therefore the magnitude of the negative charge on the G-quadruplex oligonucleotides should not shift over the range of pH values studied [80]. Therefore, our data suggests that the increase in negative zeta-potential value for G-quadruplex-loaded capsules can be attributed to negatively charged G-quadruplex oligonucleotides that can become trapped within the capsule shell after the carbonate template dissolution.

### 3.3. Enzymatic Degradation of dsDNA- and G-Quadruplex-Loaded (PMAA/PVPON)_n_ Hydrogel Capsules

**Figure 5:**
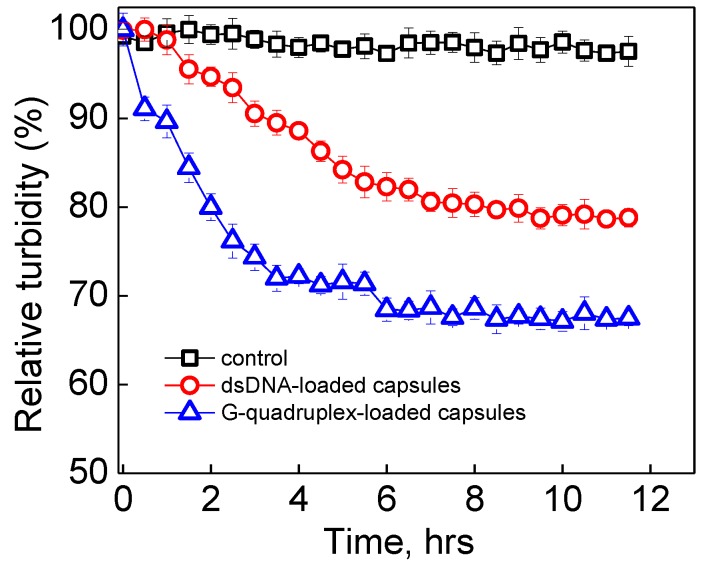
Relative solution turbidity of dsDNA-loaded (red circles), G-quadruplex DNA-loaded (blue triangles) (PMAA/PVPON)_13_ hydrogel capsules incubated with 5 mM GSH (0.01 M phosphate, pH = 7.4) at 37 °C and non-treated control empty (PMAA/PVPON)_13_ (black squares). The data is presented as mean ± SD. For each marker, five measurements within 1 min of the timepoint were averaged.

Typical intracellular concentrations of the reducing enzyme GSH have been shown to break the disulfide bond in the cystamine crosslinks as used in our study [55,56]. We have also shown that multilayer hydrogel films and particles can degrade completely after incubation with 5 mM GSH in 0.01 M phosphate buffer (pH = 7.4) at 37 °C [63]. Therefore, we studied the degradability of the (PMAA/PVPON) interpenetrated hydrogel shells in GSH solutions of the same intracellular concentration using turbidimetry. DNA-loaded (PMAA/PVPON)_13_ capsules were added to 5 mM GSH (0.01 M phosphate) at pH = 7.4 and 37 °C, and the turbidity of the solution was monitored fluorometrically at 700 nm as shown in Figure 5. It is expected that as the disulfide crosslinks between the PMAA chains are reduced to thiols, some polymers resolubilize, causing a decrease in turbidity from the insoluble capsule shell networks [63,64]. Figure 5 shows that, while the untreated (PMAA/PVPON)_13_ hydrogel capsules used as the turbidity reference control maintained their turbidity for the 12-h period (Figure 5, squares), the hydrogel capsules loaded with G-quadruplex oligonucleotides exhibited a decrease in solution turbidity of 35% over 12 h when treated with 5 mM GSH compared to the untreated capsules (Figure 5, triangles).

The incomplete decrease in solution turbidity for the G-quadruplex loaded capsules can be attributed to the presence of G-quadruplex oligomers within the capsule shell, which is in agreement with our zeta-potential measurements (Figure 4), as DNA nucleotides can participate in the binding with multilayers and stabilize the capsule shell as shown in previous works [48,49]. The DNA molecules initially entrapped into calcium carbonate cores and released into the inner capsule cavity after core dissolution can interact with the PEI priming layer, as well as with single-end reacted amine groups from the cystamine crosslinker, and because of the chain entanglement of PVPON and PMAA, the DNA molecules can additionally stabilize the capsule shell against full hydrogel degradation during crosslink reduction.

To reveal the effect of DNA molecular weight on the shell stabilization, (PMAA/PVPON)_13_ hydrogel capsules with double-stranded calf thymus DNA (dsDNA, 450 kDa) were employed under the same enzymatic conditions and the solution turbidity was measured over time (Figure 5, circles). As shown in Figure 5, the solution turbidity in this case decreased by only 20% for the dsDNA-loaded capsules, indicating that, compared to the G-quadruplex oligomers, the larger molecular weight dsDNA nucleotides could further increase the stability of the capsule shell against complete enzymatic degradation. We hypothesized that, although the capsules did not completely degrade at the typical concentration of intracellular GSH [81,82], the smaller G-quadruplex DNA could potentially escape the capsules in significant amounts in response to the treatment if the capsule shell was allowed to be thin enough. To test this hypothesis, we prepared 2-fold thinner (PMAA/PVPON)_6_ hydrogel capsules loaded with the same concentration of G-quadruplex DNA as the (PMAA/PVPON)_13_ capsules to be subjected to GSH treatment as discussed above. After extensive dialysis of the (PMAA/PVPON)_6_ capsules after core dissolution to remove calcium ions and any unbound/non-encapsulated DNA, the capsules were subjected to 5 mM GSH at pH = 7.4 (0.01 M phosphate) at 37 °C.

**Figure 6:**
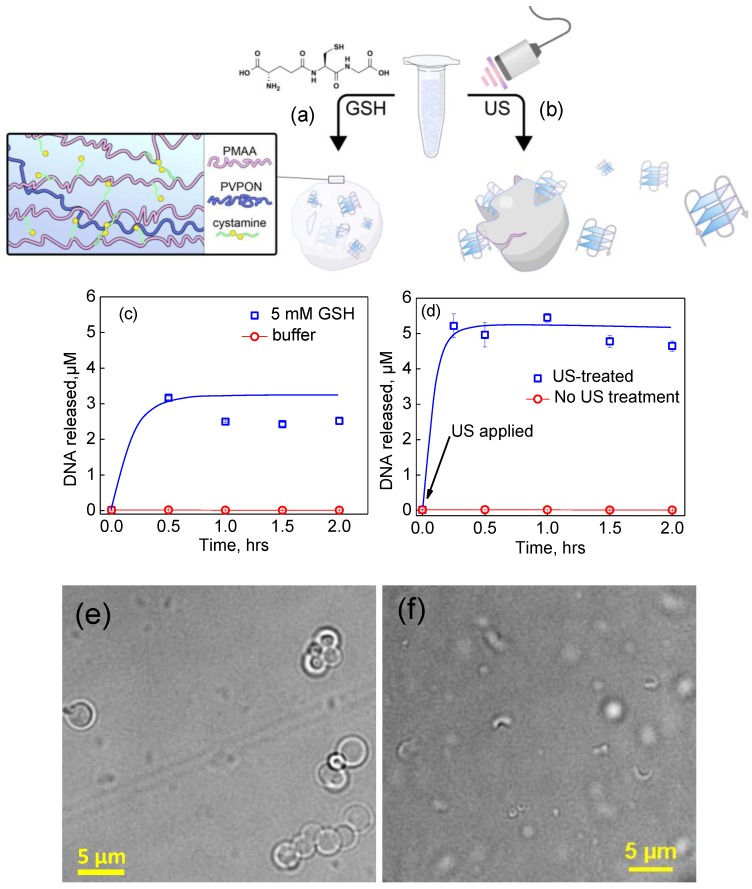
Release of DNA from (PMAA/PVPON)_6_ hydrogel capsules via (**a**) GSH-treatment and (**b**) US irradiation. Capsules treated (**c**) with 5 mM GSH at 37 °C and (**d**) with US, were pelleted via centrifugation and the supernatant was removed for quantification via UV–Vis absorbance at 260 nm. For each marker, three aliquots were taken for measurement and averaged. Optical images of (PMAA/PVPON)_6_ hydrogel capsules (**e**) before and (**f**) after US irradiation (14 W/cm^2^ for 60 s in 20-s bursts).

Remarkably, a significant concentration of G-quadruplex DNA of 3 µM for the capsules at the same concentration as the experiments discussed above was rapidly (after 30 min of exposure) released into solution in response to the enzymatic degradation (Figure 6a,c). After stabilizing at 2 h, the total concentration of released DNA was measured to be 2.52 µM, accounting for 50% of the DNA loading capacity. The slight decrease in DNA in the capsule supernatant observed over the next 1.5 h can be attributed to some amounts of the DNA readsorbing on to partially degraded hydrogel shells via interaction with the PEI priming layer. This data demonstrates that enzymatic degradation can offer a viable release pathway for delivery of G-quadruplex oligonucleotides from the (PMAA/PVPON) interpenetrated hydrogel capsules.

### 3.4. Ultrasound (US)-Triggered Release of G-Quadruplex DNA from (PMAA/PVPON)_n_ Hydrogel Capsules

Because of the incomplete release of oligonucleotides from the G-quadruplex DNA-loaded (PMAA/PVPON) hydrogel capsules due to the stabilization of the capsule shell via its interactions with the DNA molecules, we studied the ability of the (PMAA/PVPON) hydrogel capsules to release G-quadruplex DNA upon US irradiation (Figure 6b). G-quadruplex DNA-loaded (PMAA/PVPON)_6_ were exposed to 22 kHz US at 14 W/cm^2^ for 60 s in 20-s bursts. After the exposure, the supernatant of each capsule solution was monitored using UV–Vis spectroscopy to calculate the concentration of G-quadruplex DNA released per capsule in the sample, as optical microscopy revealed that the US treatment had quantitatively destroyed the capsules (Figure 6e,f). This result is in agreement with the observation we made in a previous study that the mechanical force of this 22 kHz ultrasound irradiation is sufficient to completely destroy our multilayer microcapsules [65]. For the (PMAA/PVPON)_6_ capsules, we found that approximately 37 attomoles of G-quadruplex oligonucleotides were released per capsule (4.95 µM at 1 mL of 1.6 × 10^8^ capsules/mL) (Figure 6c). As the US irradiation completely destroyed all capsules in solution, rendering them into small fragments, the released DNA that was not readsorbed onto the polymeric complex fragments was taken as the effective loading capacity, i.e., the highest amount of DNA that could be released from the capsules. This value was used to calculate the % release of DNA resulting from the GSH treatment as discussed above. Previously, double- and single-stranded DNA was encapsulated within PMAA hydrogel capsules prepared using porous silica spheres as the core template [83]. In that study, it was found that the upper limit to the number of 20-mer ssDNA strands that could be adsorbed to the surface of the silica template, and thus become encapsulated, approached 10,000 chains per capsule. In contrast, we show a significant increase in the number of 24-mer G-quadruplex oligonucleotide chains that can be loaded per capsule, representing about a 2000-fold increase in encapsulated DNA by utilizing the entire volume of the core during DNA co-precipitation.

**Figure 7:**
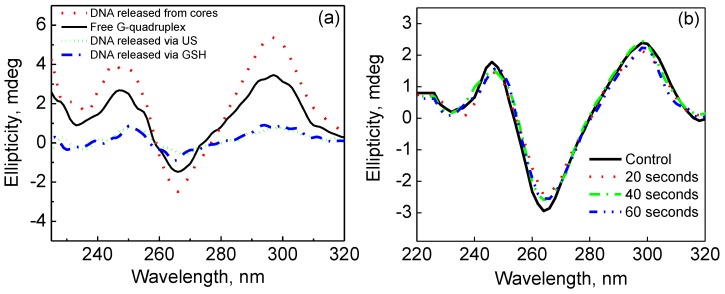
CD spectra of free G-quadruplex oligonucleotides (solid black) after encapsulation and release from the CaCO_3_ cores via dissolution in EDTA (dot red) or after release from the (PMAA/PVPON)_13_ capsules via degradation by GSH (dash dot blue) or 22 kHz ultrasound (55 W/cm^2^ for 20 s) (short dot green). G-quadruplex oligonucleotides (solid black) were exposed to 22 kHz ultrasound (55 W/cm^2^) for various times and measured for retention of secondary structure.

Importantly, the sonoporation effect of US irradiation is accompanied by microbubble cavitation that can cause local heating and high mechanical pressure [33]. In this regard, it is important that the US pressure used to release encapsulated nucleotides does not have harmful effects on the structure of the DNA. Especially in the case of G-quadruplex forming sequences, where the secondary structure of the DNA plays a crucial role in its function [13], ensuring that the stacked tetrad conformation is intact after encapsulation and release is essential in promoting the efficacy of the delivery system as a therapeutic strategy. Therefore, to determine if the encapsulation strategy and US-triggered release used in this work was suitable for G-quadruplex delivery, we first studied the effect of coprecipitation and core dissolution on the structure of the G-quadruplex oligonucleotides using circular dichroism (CD) spectroscopy (Figure 7a).

A solution of free G-quadruplex oligonucleotide was used as a positive control and the characteristic peaks and trough (245, 295, and 265 nm, respectively) for a G-quadruplex basket structure [84] can be seen in Figure 7a (solid black line). The spectrum in Figure 7a corresponding to coprecipitated and released G-quadruplex (Figure 7a, red dot line) shows the same characteristic peaks and trough as the free control (Figure 7a, solid black line), which indicates that trapping the nucleotide within the crystalline CaCO_3_ network and subsequently releasing it by dissolving the precipitated particles in EDTA solution did not alter the secondary structure of the G-quadruplex DNA sequence. Similar results were also found for G-quadruplex oligonucleotides released from (PMAA/PVPON) capsules via US irradiation (Figure 7a, short dot green line). In addition, the CD spectrum of G-quadruplex DNA released from capsules via GSH-triggered crosslink reduction has the same features as the former ones (Figure 7a, dash dot blue line).

All samples of released DNA were found to retain their secondary structure as indicated by the characteristic peaks that match that of the free G-quadruplex DNA control (Figure 7a). To demonstrate the robust nature of the G-quadruplex stacked tetrads, solutions of free G-quadruplex oligonucleotides were exposed to US of 55 W/cm^2^ for 20, 40, or 60 s and were monitored using circular dichroism to detect any changes to the secondary structure of the G-quadruplex (Figure 7b). After US exposure, the characteristic basket conformation peaks for a G-quadruplex stack were observed for all solutions (Figure 7b). The lack of change to the secondary structure for US-exposed nucleotides indicates that the G-quadruplex is stable enough to undergo US treatment totaling at least 55 W/cm^2^ power intensity (almost three times the power applied in our release studies for PMAA/PVPON hydrogel capsules) for up to 60 s and remain in its folded state. This is in good agreement with previous reports on the effects of sonication on G-quadruplex structure [84]. Our data shows that our encapsulation strategy, enzymatic degradation approach, and, most importantly, our US-triggered release of G-quadruplex from the (PMAA/PVPON)_n_ hydrogel capsules represents a viable pathway to controlled delivery of the nucleotide that stands up against any potentially harmful effects of the delivery method.

### 3.5. Encapsulation and US-Triggered Release of dsDNA from (PMAA/PVPON)_13_ Capsules

**Figure 8:**
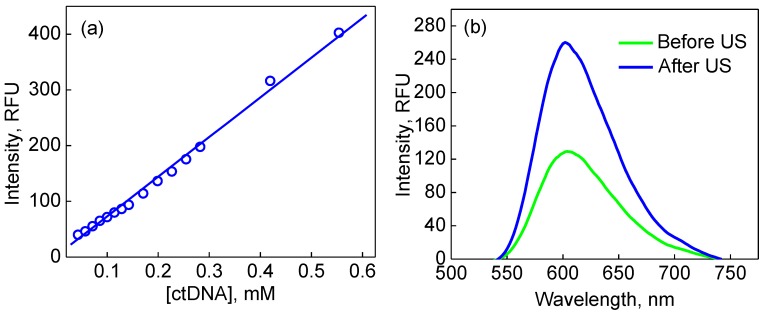
(**a**) dependence of fluorescence of dsDNA intercalated with EtBr on dsDNA concentration; (**b**) release of dsDNA from (PMAA/PVPON)_13_ capsules after US-treatment with 22 kHz US (55 W/cm^2^ for 20 s) as quantified with fluorescence microscopy.

To explore the versatility of the (PMAA/PVPON)_n_ interpenetrated hydrogel capsules and demonstrate the potential of US-triggered release from the hydrogel capsules, we also explored this system for encapsulation and release of dsDNA with a molecular weight 60 times larger than that of the G-quadruplex oligonucleotides. As summarized in Figure 1, dsDNA was coprecipitated within CaCO_3_ particles followed by assembly of 13 bilayers of PMAA/PVPON, which represents the system with the highest resistance to mechanical pressure among those that we have used in the current study. Ethidium bromide (EtBr), a well-known DNA intercalator [85], was used as a fluorescent label in the release studies. We examined the ability of our capsules to release dsDNA upon US exposure as described earlier for the release of G-quadruplex oligonucleotides. Solutions of dsDNA-loaded (PMAA/PVPON)_13_ hydrogel capsules were exposed to US at 55 W/cm^2^ power intensity for 20 s and the supernatant was examined for the presence of dsDNA released from the capsules via fluorescence measurements. As the molar extinction coefficient of dsDNA is much lower than that of the G-quadruplex oligomers, (6600 M^−1^·cm^−1^ versus 228,500 M^−1^·cm^−1^, respectively) [86], UV–Vis spectroscopy was not useful in quantifying the released DNA. To solve this, the binding of EtBr to the dsDNA and subsequent linear relationship [85] in fluorescence spectroscopy was utilized as a way to monitor the amount of dsDNA present in the supernatant (Figure 8a). We found that a suspension of 1.6 × 10^8^ capsules/mL, i.e., the same concentration of capsules as used in the G-quadruplex release studies, resulted in an US-triggered release of dsDNA totaling 180 µM (Figure 8b). The release data for dsDNA shows that the (PMAA/PVPON) interpenetrated hydrogel capsules may serve as a generalizable encapsulation and delivery vehicle for nucleotides of a wide range of molecular weights and functions.

## 4. Conclusions

New and promising roles for nucleic acid therapeutics have been emerging in recent studies. However, challenges with delivery and efficiency have limited their prospects for translation. We address the need for improved delivery vehicles of nucleic acids in this work through the development of multilayer capsules made of interpenetrated hydrogel of (PMAA/PVPON) that can be used for the encapsulation and release of various DNA structures including G-quadruplex and double-stranded helices. We show that (PMAA/PVPON)_n_ capsules loaded with either G-quadruplex or dsDNA have a higher effective loading capacity compared to previous studies, decreasing the number of capsules required per dose. In addition, we show that these capsules are responsive toward both enzymatic degradation by intracellular concentration of the reducing enzyme GSH and US exposure, allowing for precisely controlled delivery of the DNA cargo. These hydrogel capsules represent a significant advancement in the potential of microparticle drug delivery agents that enable the most desirable properties of gene-based therapeutics and may help bridge the gap toward realizing the next generation of life saving therapeutic strategies for disease treatment.
